# Common and specific gene signatures among three different endometriosis subtypes

**DOI:** 10.7717/peerj.8730

**Published:** 2020-03-05

**Authors:** Li Jiang, Mengmeng Zhang, Sixue Wang, Yuanyuan Han, Xiaoling Fang

**Affiliations:** 1 Department of Obstetrics and Gynecology, The Second Xiangya Hospital, Central South University, Changsha, Hunan, China; 2 Center of Tree Shrew Germplasm Resources, Institute of Medical Biology, Chinese Academy of Medical Sciences and Peking Union Medical College, Kunming, Yunnan, China; 3 Morning Star Academic Cooperation, Shanghai, China

**Keywords:** Bioinformatic analysis, Differentially expressed genes, Microarray, Endometriosis, Subtype

## Abstract

**Aims:**

To identify the common and specific molecular mechanisms of three well-defined subtypes of endometriosis (EMs): ovarian endometriosis (OE), peritoneal endometriosis (PE), and deep infiltrating endometriosis (DIE).

**Methods:**

Four microarray datasets: GSE7305 and GSE7307 for OE, E-MTAB-694 for PE, and GSE25628 for DIE were downloaded from public databases and conducted to compare ectopic lesions (EC) with eutopic endometrium (EU) from EMs patients. Differentially expressed genes (DEGs) identified by limma package were divided into two parts: common DEGs among three subtypes and specific DEGs in each subtype, both of which were subsequently performed with the Kyoto Encyclopedia of Genes (KEGG) pathway enrichment analysis. The protein-protein interaction (PPI) network was constructed by common DEGs and five hub genes were screened out from the PPI network. Besides, these five hub genes together with selected interested pathway-related genes were further validated in an independent OE RNA-sequencing dataset GSE105764.

**Results:**

A total of 54 EC samples from three EMs subtypes (OE, PE, DIE) and 58 EU samples were analyzed, from which we obtained 148 common DEGs among three subtypes, and 729 specific DEGs in OE, 777 specific DEGs in PE and 36 specific DEGs in DIE. The most enriched pathway of 148 shared DEGs was arachidonic acid (AA) metabolism, in which most genes were up-regulated in EC, indicating inflammation was the most common pathogenesis of three subtypes. Besides, five hub genes AURKB, RRM2, DTL, CCNB1, CCNB2 identified from the PPI network constructed by 148 shared DEGs were all associated with cell cycle and mitosis, and down-regulated in EC, suggesting a slow and controlled proliferation in ectopic lesions. The KEGG pathway analysis of specific DEGs in each subtype revealed that abnormal ovarian steroidogenesis was a prominent feature in OE; OE and DIE seems to be at more risk of malignant development since both of their specific DEGs were enriched in the pathways in cancer, though enriched genes were different, while PE tended to be more associated with dysregulated peritoneal immune and inflammatory microenvironment.

**Conclusion:**

By integrated bioinformatic analysis, we explored common and specific molecular signatures among different subtypes of endometriosis: activated arachidonic acid (AA) metabolism-related inflammatory process and a slow and controlled proliferation in ectopic lesions were common features in OE, PE and DIE; OE and DIE seemed to be at more risk of malignant development while PE tended to be more associated with dysregulated peritoneal immune and inflammatory microenvironment, all of which could deepen our perception of endometriosis.

## Introduction

Endometriosis (EMs), characterized by the growth of endometrium-type tissue outside the uterine cavity, is a common and usually chronic (long-term) inflammatory disorder, affecting 5–10% of women in their reproductive years (Zondervan et al., 2018). EMs is also considered as a phenotypically heterogeneous condition not only due to diverse symptoms, such as infertility, pelvic pain or dysmenorrhea but also different lesion locations, predominantly but not exclusively, in the pelvic compartment (Vercellini et al., 2014). Since the classic retrograde menstruation hypothesis (Sampson, 1927) that during menstrual uterine contractions, endometrial fragments via trans-tubal reflux flowed to implant onto the peritoneum and abdominal organs could not explain the fact that 76–90% of women experienced retrograde menstruation but not all of these women suffered from EMs (Blumenkrantz et al., 1981), there must exist other mechanisms facilitating the development of EMs. On the other hand, as early as 1997, Nisolle and Donnez provided morphological and histochemical evidence indicating that three main subtypes of endometriosis: ovarian endometriosis (OE), peritoneal endometriosis (PE), and deep infiltrating endometriosis (DIE), should be considered different entities, though they shared the histologic features of endometrial glands and stroma (Nisolle & Donnez, 1997). Thus, investigating the common and specific mechanisms among different EMs subtypes may provide new insight into the pathogenesis of endometriosis. However, due to the limited information as well as samples available from single cohorts, few integrative analyses of EMs subtypes were conducted.

**Figure 1:**
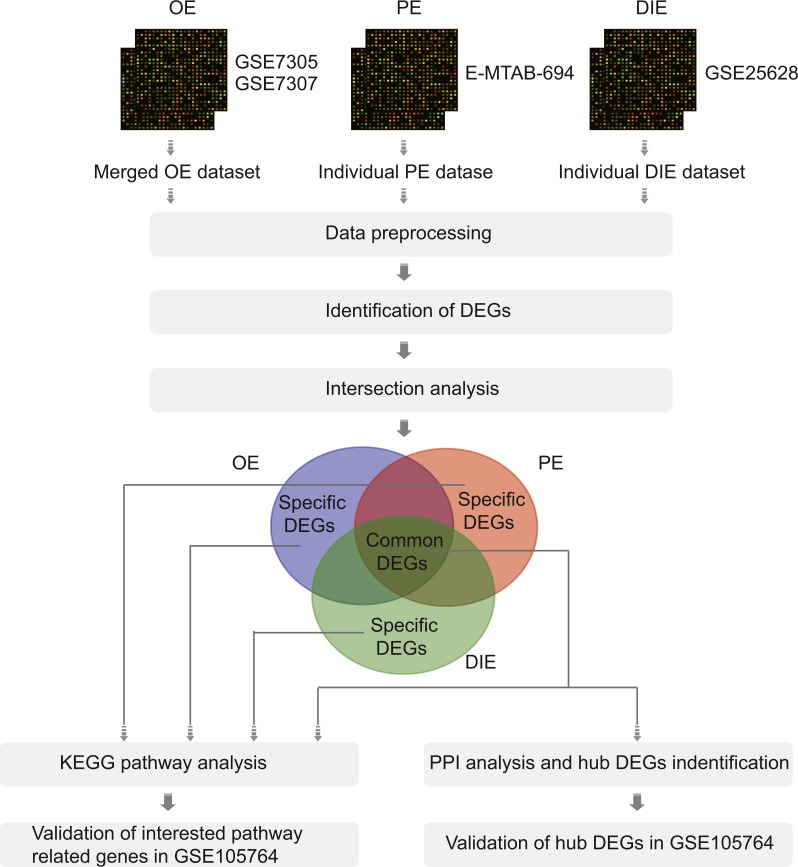
The flowchart of integrative analysis of microarray datasets from three different endometriosis subtypes. Abbreviations: OE, ovarian endometriosis; PE, peritoneal endometriosis; DIE, deep infiltering endometriosis; DEGs, differentially expressed genes; KEGG, KyotoEncyclopedia of Genes; PPI, protein-protein interaction.

Therefore, in this article, we analyzed the microarray datasets GSE7305 and GSE7307 of OE, E-MTAB-694 of PE, and GSE25628 of DIE to obtain common differentially expressed genes (DEGs) among three EMs subtypes along with specific DEGs in each subtype by comparing ectopic lesions (EC) with eutopic endometrium (EU) from EMs patients. Then, the Kyoto Encyclopedia of Genes (KEGG), protein-protein interaction (PPI) network and validation analysis were performed to analyze these common and specific DEGs (Fig. 1). Overall, all results were combined to promote further understanding of different EMs subtypes and reveal a more thorough landscape of EMs.

## Materials & Methods

### Data resources

The search for endometriosis-related microarray datasets was conducted in two public databases: Gene Expression Omnibus (https://www.ncbi.nlm.nih.gov/geo/) and Array-Express (https://www.ebi.ac.uk/ar-rayexpress/). The keywords: ‘endometriosis’, ‘endometrium’, ‘tissue’, ‘homo sapiens’ or ‘human’ respectively with ‘ovarian’, ‘peritoneal’, ‘DIE’, ‘deep’ or ‘infiltrating’ were employed to mine the datasets for three EMs subtypes. Additionally, all selected datasets were based on Affymetrix platforms to reduce the ‘platform effect’ due to different probe designs among different companies. Finally, four datasets were included: GSE7305 and GSE7307 for OE, E-MTAB-694 for PE, and GSE25628 for DIE (Table 1), all of which contained at least 8 samples for both ectopic lesions (EC) and eutopic endometrium (EU) from the EMs patients. A total of 54 EC samples from these datasets of different EMs subtypes and 58 EU samples were included.

**Table 1:** Basic information of the microarray datasets of three different EMs subtypes.

**Subtype**	**Accession**	**Platform**	**No. of probes**	** No. of samples (EU/EC, whether paired)**	**Tissue stage**	**References**
OE	GSE7305	GPL570 [HG-U133_Plus_2]	54,675	20 (10/10, paired)	2 Follicular phases and 8 Luteal phases	Hever et al. (2007)
	GSE7307	GPL570 [HG-U133_Plus_2]	54,675	41 (23/18, unknown)	Unpublished	Unpublished
PE	E-MTAB-694	GPL570: [HG-U133_Plus_2]	54,675	35 (17/18, most were paired)	8 Proliferative phases and 9 Secretory phases	Sohler et al. (2013)
DIE	GSE25268	GPL571: [HG-U133A_2]	22,277	16 (08/08, paired)	16 Proliferative phases	Crispi et al. (2013)

**Notes.**

EMs endometriosis OE ovarian endometriosis PE peritoneal endometriosis DIE deep infiltrating endometriosis EU eutopic endometrium EC ectopic lesions

Besides, all EC and EU samples in GSE7305 and GSE25628, as well as most in E-MTAB-694, were paired, meaning they were obtained from the same patients. The information on patients’ clinical characteristics in E-MTAB-694 for PE and GSE25628 for DIE were provided in the Tables S1 and S2 according to the original articles (Crispi et al., 2013; Sohler et al., 2013). However, the clinical information of patients was unavailable in the two datasets for OE: GSE7305 (not provided by the original article (Hever et al., 2007)) and GSE7307 (not originally published). Since our analysis did not involve any experiment on humans or animals directly, ethical approval was not necessary.

### Data preprocessing

The raw data CEL files and group information of four datasets were downloaded from the GEO and Array-Express database. Firstly, the CEL files of GSE7305 and GSE7307 of subtype OE were combined into an integrated dataset, which was subsequently named the ‘merged OE dataset’. Then, CEL files of this ‘merged OE dataset’ as well as the individual PE dataset E-MTAB-694 and DIE dataset GSE25628 were read by ‘Affy’ R package (Gautier et al., 2004), and further processed by the robust multi-array average (RMA) method for background correction, normalization, and expression calculation (Irizarry et al., 2003). Moreover, the ‘ComBat’ function in the SVA R package (Leek et al., 2012) was utilized to adjust the batch effects in the merged OE dataset, and then the principal component analysis (PCA) was applied to assess the performance of the batch effect adjustment. Also, the individual PE and DIE datasets were performed with PCA analysis to visualize sample distributions. Furthermore, probes were annotated according to the annotation files provided by Affymetrix official website (http://www.affymetrix.com/), and unmatched probes were abandoned.

### Identification of DEGs

After pretreatment, R package limma (Ritchie et al., 2015) was applied to filtrate DEGs of these three datasets: the merged OE dataset of GSE7305 and GSE7307, the individual PE dataset E-MTAB-694 and DIE dataset GSE25628, respectively, with the cutoff criteria: —log_2_ fold change (FC)—>1 and *p*-value <0.05. Additionally, the intersection analysis was conducted among three sets of DEGs.

### KEGG pathway enrichment analysis

To explain common pathogenesis among three EMs subtypes (OE, PE, and DIE), KEGG pathway enrichment analysis of shared DEGs among the merged OE dataset of GSE7305 and GSE7307, the individual PE dataset E-MTAB-694 and DIE dataset GSE25628 was performed by DAVID (http://david.abcc.ncifcrf.gov/), an online tool for comprehensive functional annotation of genes and proteins. Furthermore, to explore specific pathogenesis of these three subtypes, specific DEGs in each dataset were also conducted with KEGG pathway enrichment analyses by DAVID. A threshold of *p*-value <0.05 was utilized to filter all KEGG pathways, which were also ranked by the *p*-value.

### Validation of interested pathway-related genes in GSE105764

Considering all training datasets were comprised of microarray data, we intended to validate interested pathways related genes, for example, the most enriched pathway of common DEGs or representative pathways in the certain subtype, in RNA-sequencing datasets. However, due to the lack of PE and DIE RNA-sequencing datasets in searching results, we only performed the validation analysis in an OE RNA-sequencing dataset GSE105764 from the GEO database, which contained 8 paired EC and EU tissue samples based on GPL20301 (Illumina HiSeq 4000) (Zhao et al., 2018). The raw read counts were calculated by the DEseq2 R package (Love, Huber & Anders, 2014) to obtain the DEGs. Additionally, we also listed the expression of selected genes in the previously analyzed results of microarray datasets as a comparison.

### PPI networks construction and identification of hub DEGs

The identified common DEGs among three EMs subtypes were uploaded to the online database STRING (http://string-db.org/; version 11.0) to explore their interactions at the protein level, with an interaction score >0.4 as the cutoff value. Afterward, the PPI network was visualized in software Cytoscape (version 3.6.1) and analyzed by the ‘Degree method’ in plugin Cytohubba to identify the top 5 hub nodes in the network.

### Validation analysis of hub DEGs in GSE105764

We also performed the validation analysis of the top 5 hub DEGs identified from the PPI network constructed of 148 common DEGs among three EMs subtypes in the OE RNA-sequencing dataset GSE105764. Additionally, we also listed the expression of these hub genes in the previously analyzed results of microarray datasets as a comparison.

## Results

### Data preprocessing and Identification of DEGs

**Figure 2:**
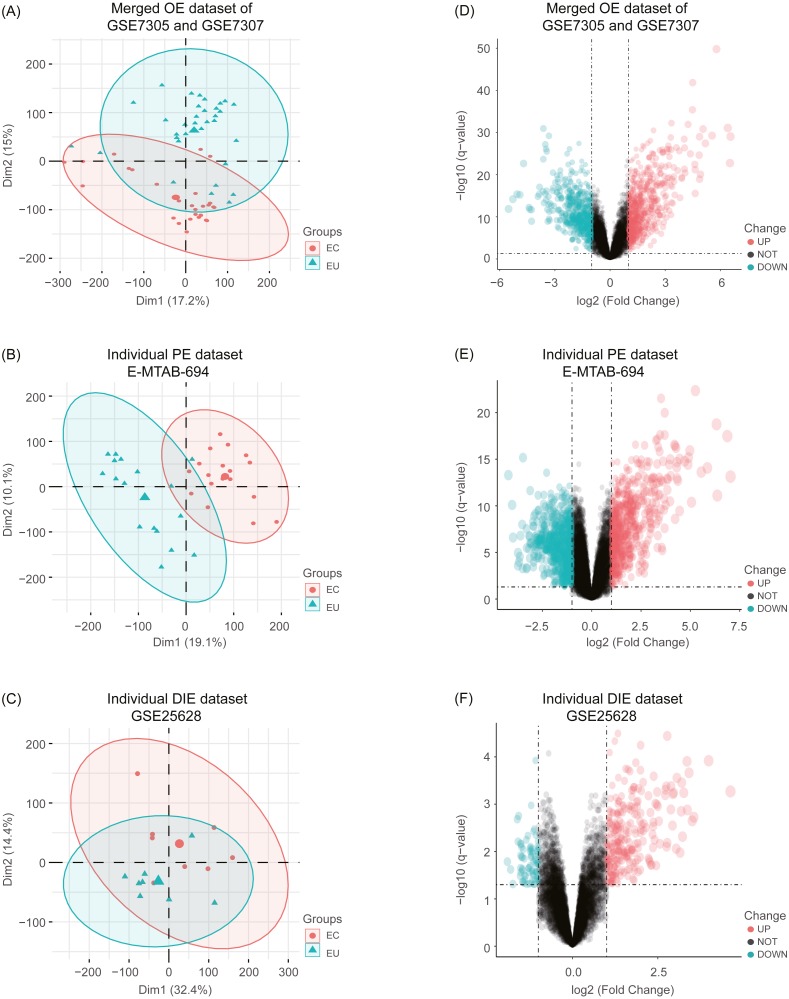
Principal component analysis (PCA) and identification of differentially expressed genes (DEGs) in each EMs subtype dataset. (A), (B), (C) After normalization of raw data in the merged OE dataset of GSE7305 and GSE7307, the individual PE dataset E-MTAB-694 and the DIE dataset GSE25628, the principal component analysis (PCA) was performed to examine the differences between EC and EU groups; (D), (E), (F) Volcano plots of the distributions of DEGs between EC and EU in the merged OE dataset of GSE7305 and GSE7307, the individual PE dataset E-MTAB-694 and the DIE dataset GSE25628. DEGs with log_2_FC >1 were shown in red dots; DEGs with log_2_FC < − 1 were in green dots (*P* < 0.05). No significantly changed genes are marked as black dots. EMs, endometriosis; OE, ovarian endometriosis; PE, peritoneal endometriosis; DIE, deep infiltrating endometriosis; EC, ectopic lesions; EU, eutopic endometrium; log_2_FC, log_2_ Fold Change.

After combing the expression data, the merged OE dataset of GSE7305 and GSE7307 included 33 EU and 28 ovarian EC samples. Besides, the individual PE dataset E-MTAB-694 contained 17 EU and 18 peritoneal EC samples, and the individual DIE dataset GSE25628 comprised 8 EU and 8 deep infiltrating EC samples. After normalization of raw data in these datasets, boxplots were depicted, showing even mean values of gene expression in each sample. (Fig. S1). Moreover, the principal component analysis (PCA) was performed to examine the differences between EC and EU groups (Figs. 2A, 2B and 2C).

**Figure 3:**
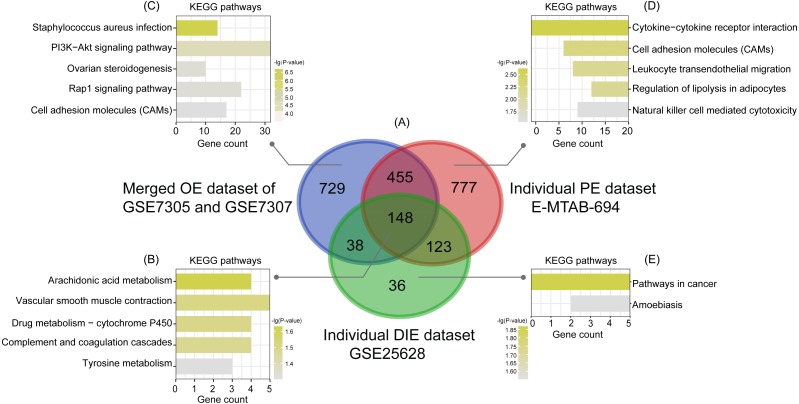
Intersection analysis and KEGG pathway enrichment analysis of common and specific DEGs among OE, PE, and DIE. (A) Venn diagram of DEGs selected with —log_2_FC—>1 and *p*-value < 0.05 from the merged OE dataset of GSE7305 and GSE7307, the individual PE dataset E-MTAB-694 and the individual DIE dataset GSE25628. These three datasets showed an overlap of 148 genes; (B) significantly enriched KEGG pathways of 148 shared DEGs; (C) significantly enriched KEGG pathways of 729 DEGs specific to the merged OE dataset of GSE7305 and GSE7307; (D) significantly enriched KEGG pathways of 777 DEGs specific to the individual PE dataset E-MTAB-694; (E) significantly enriched KEGG pathways of 36 DEGs specific to the individual DIE dataset GSE25628. All the entries were screened out with the *p*-value <0.05 and ranked by *p*-value.

Subsequently, analyzed by the R package limma (Ritchie et al., 2015) with the threshold of —log_2_ FC— >1 and *p*-value <0.05, a total of 744 upregulated as well as 626 downregulated DEGs were obtained from the merged OE dataset of GSE7305 and GSE7307, along with 731 upregulated and 772 downregulated DEGs from the individual PE dataset E-MTAB-694, and 268 upregulated and 77 downregulated DEGs from the individual DIE dataset GSE25628. Volcano plots were depicted for the visualization of DEGs in each dataset (Figs. 2D, 2E and 2F). The intersection analysis indicated that 148 genes were common to three datasets, 729 DEGs specific to the merged OE dataset, 777 DEGs specific to the individual PE dataset and only 36 DEGs specific to the individual DIE dataset owing to relatively fewer probes in this dataset, as shown in the Venn diagram (Fig. 3A).

### KEGG enrichment analysis of common and specific DEGs

Based on the KEGG analysis from the online tool DAVID, 148 common DEGs were mainly enriched in the pathways of arachidonic acid (AA) metabolism, vascular smooth muscle contraction, drug metabolism-cytochrome P450, complement and coagulation cascades, tyrosine metabolism, according to the *p*-value ranking (Fig. 3B).

**Table 2:** The top five enriched KEGG pathways of common DEGs among three EMs subtypes datasets and specific DEGs in each EMs subtype dataset.

**Category of DEGs**	**KEGG ID**	**Pathway description**	**Count**	***P*-value**	**Enriched Genes**
**148 common DEGs among the merged OE dataset of GSE7305 and GSE7307, the individual PE dataset E-MTAB-694 and DIE dataset GSE25628**	hsa00590	Arachidonic acid metabolism	4	0.023854965	GGT5, CYP2J2, PTGIS, PLA2G2A
	hsa04270	Vascular smooth muscle contraction	5	0.030857192	ACTG2, AGTR1, ACTA2, PLA2G2A, MYL9
	hsa00982	Drug metabolism - cytochrome P450	4	0.031595883	FMO1, FMO2, AOX1, ADH1B
	hsa04610	Complement and coagulation cascades	4	0.032797081	C7, THBD, C3, CFH
	hsa00350	Tyrosine metabolism	3	0.048956335	AOX1, ADH1B, AOC3
**729 specific DEGs in the merged OE dataset of GSE7305 and GSE7307**	hsa05150	Staphylococcus aureus infection	14	2.19096E-07	HLA-DQB1, ICAM1, C3AR1, C5AR1, MASP1, CFB, C1R, ITGB2, C1S, C1QC, HLA-DQA1, FCGR2C, FCGR2A, C2
	hsa04151	PI3K-Akt signaling pathway	32	4.06007E-05	FGFR2, FGFR3, OSMR, FGF9, TNC, ITGA11, FGF13, KIT, LPAR1, ITGB8, IL4R, COL27A1, PIK3AP1, ANGPT1, GNG2, GNG4, THBS1, COL11A1, INSR, PPP2R2C, PIK3R1, FN1, MET, NR4A1, IGF1, CCND1, CDKN1A, ITGA6, PRLR, VEGFA, PDGFRA, JAK3
	hsa04913	Ovarian steroidogenesis	10	0.00016359	CYP17A1, PLA2G4A, PTGS2, CYP11A1, STAR, IGF1, SCARB1, ALOX5, INSR, BMP6
	hsa04015	Rap1 signaling pathway	22	0.000172943	FGFR2, FGFR3, FGF9, MET, SIPA1L2, IGF1, ITGB2, FGF13, LPAR1, KIT, DOCK4, RASSF5, PLCB4, ID1, VEGFA, PDGFRA, RAPGEF5, ANGPT1, THBS1, PLCB1, INSR, PIK3R1
	hsa04514	Cell adhesion molecules (CAMs)	17	0.00026996	HLA-DQB1, ICAM1, PTPRC, MAG, CLDN4, CADM1, ITGB2, CLDN11, CDH3, HLA-DQA1, CD86, ITGA6, ITGB8, CNTN1, CNTNAP2, VCAN, JAM3
**777 specific DEGs in the individual PE dataset E-MTAB-694**	hsa04060	Cytokine-cytokine receptor interaction	21	0.002513955	CXCL1, TNFRSF21, IL2RB, CSF1, CXCL3, IL19, LIFR, CD40, CCL4, LEP, LIF, CCL13, TNFRSF10B, PPBP, CCL20, CXCR4, CCR2, TNFRSF18, CSF2RB, BMPR1B, CSF1R
	hsa04514	Cell adhesion molecules (CAMs)	14	0.005814763	F11R, NRXN3, SELL, ICAM2, L1CAM, CD40, ITGA4, HLA-E, CDH5, SIGLEC1, SDC1, CLDN1, ESAM, CD28
	hsa04670	Leukocyte transendothelial migration	12	0.00786069	F11R, EZR, VAV3, CXCR4, MAPK13, CLDN1, ESAM, TXK, ITGA4, CDH5, PRKCB, VCL
	hsa04923	Regulation of lipolysis in adipocytes	8	0.007916063	ADCY4, ADRB2, PLIN1, ADCY5, NPY1R, PRKG1, TSHR, LIPE
	hsa04650	Natural killer cell-mediated cytotoxicity	11	0.029482747	CD48, PRF1, VAV3, TNFRSF10B, ICAM2, KIR2DS2, GZMB, KIR2DL3, KIR2DL2, KIR2DL4, PRKCB
**36 specific DEGs in the individual DIE dataset GSE25628**	hsa05200	Pathways in cancer	5	0.013783298	COL4A3, PTGER3, COL4A6, RAD51, F2R
	hsa05146	Amoebiasis	3	0.027515834	COL4A3, ARG2, COL4A6

**Notes.**

KEGG Kyoto Encyclopedia of Genes DEGs differentially expressed genes EMs endometriosis OE ovarian endometriosis PE peritoneal endometriosis DIE deep infiltrating endometriosis

Likewise analyzed by DAVID, KEGG analysis showed that the top5 significantly enriched pathways of 729 specific DEGs in the merged OE dataset of GSE7305 and GSE7307 were staphylococcus aureus infection, PI3K-Akt signaling pathway, ovarian steroidogenesis, Rap1 signaling pathway, and cell adhesion molecules (CAMs) (Fig. 3C); the top5 significantly enriched pathways of 777 specific DEGs in the individual PE dataset E-MTAB-694 were cytokine-cytokine receptor interaction, cell adhesion molecules (CAMs), leukocyte trans-endothelial migration, regulation of lipolysis in adipocytes, and natural killer cell-mediated cytotoxicity (Fig. 3D); 36 specific DEGs in the individual DIE dataset GSE25628 were only enriched in two pathways: pathways in cancer and amoebiasis (Fig. 3E). Furthermore, the above-mentioned significant enriched pathways related genes were detailed in Table 2.

### Validation of interested pathway-related genes in GSE105764

Since endometriosis was defined as an inflammatory disorder (Bulun et al., 2019) and the top 1 enriched pathway of 148 common DEGs was the arachidonic acid (AA) metabolism pathway, a key pro-inflammatory pathway (Kuehl & Egan, 1980), we validated this pathway-related genes: GGT5, CYP2J2, PTGIS and PLA2G2A in the OE RNA-sequencing dataset GSE105764. The consistency results between microarray training datasets and the RNA-sequencing validation dataset showed that most of these genes were found to be significantly higher in EC tissue than EU endometrium, except for CYP2J2 (Table 3).

**Table 3:** The expression of the arachidonic acid metabolism pathway-related genes in microarray training datasets and the OE RNA-sequencing validation dataset GSE105764.

**Gene Symbol**	**Gene Description**	**OE RNA-sequencing dataset GSE105764**		**The merged OE dataset of GSE7305 and GSE7307**		**The individual PE dataset E-MTAB-694**		**The individual DIE dataset GSE25628**	
		**Log2FC**	***P*-Value**	**Log2FC**	***P*-Value**	**Log2FC**	***P*-Value**	**Log2FC**	***P*-Value**
PTGIS	Prostaglandin I2 Synthase	6.289598638	2.43946E-44	4.631282825	2.91622E-16	4.971203942	1.20213E-16	3.508766958	0.002253242
PLA2G2A	Phospholipase A2 Group IIA	2.822326237	0.001074114	4.849610555	2.38055E-25	3.551202751	3.01372E-06	3.152761244	0.010549907
GGT5	Gamma-Glutamyltransferase 5	2.682418539	1.54252E-12	1.03319199	1.421E-13	1.031785388	5.90453E-07	1.32309443	0.006376396
CYP2J2	Cytochrome P450 Family 2 Subfamily J Member 2	−6.239846138	1.75094E-27	−1.142837781	1.79003E-09	−1.850912918	9.66805E-09	−1.160485084	0.02902089

**Notes.**

Log2 FC Log2 fold change OE ovarian endometriosis PE peritoneal endometriosis DIE deep infiltrating endometriosis

Moreover, besides the common pathogenetic pathways, each different subtype of EMs might possess its peculiar molecular signature. Since the validation dataset GSE105764 was an OE dataset and the inflammation in EMs were driven by estradiol (Bulun et al., 2019), we chose ovarian steroidogenesis—one representative pathway in subtype OE related genes to validate. The validation results showed that most genes in this pathway were significantly overexpressed in EC in subtype OE, almost consistent with that in the OE microarray training dataset, whereas with no significant difference between EU and EC in the individual PIE and DIE dataset (Table 4), which suggested ectopic tissue specificity and focal prominent aberrant hormonal environment in OE.

**Table 4:** The expression of one representative pathway in subtype OE–ovarian steroidogenesis related genes in microarray training datasets and the OE RNA-sequencing validation dataset GSE105764.

**Gene Symbol**	**Gene Description**	**OE RNA-sequencing dataset GSE105764**		**The merged OE dataset of GSE7305 and GSE7307**		**The individual PE dataset E-MTAB-694**		**The individual DIE dataset GSE25628**	
		**Log2FC**	***P*-Value**	**Log2FC**	***P*-Value**	**Log2FC**	***P*-Value**	**Log2FC**	***P*-Value**
STAR	Steroidogenic Acute Regulatory Protein	3.844497923	3.67398E-10	5.549409967	5.35385E-30	−0.691371832	0.030315396	0.2446853	0.854991
BMP6	Bone Morphogenetic Protein 6	2.585401261	9.63142E-08	2.13686869	3.12026E-22	0.16812685	0.530786767	0.3323532	0.5156626
CYP11A1	Cytochrome P450 Family 11 Subfamily A Member 1	2.571939702	3.06172E-11	1.977665697	1.63051E-09	−0.074703419 0.584259769	−0.3281747	0.7447965
SCARB1	Scavenger Receptor Class B Member 1	2.264263509	2.98276E-21	1.74100742	1.5326E-11	0.163414061	0.342902499	0.06680135	0.9171244
ALOX5	Arachidonate 5-Lipoxygenase	1.932324569	2.01964E-06	1.113589966	2.23627E-05	−0.030632071	0.87312448	0.2211488	0.7237545
PTGS2	Prostaglandin-Endoperoxide Synthase 2	1.499535863	0.021110249	1.188644849	0.000423004	−1.324453675 0.132193434	−0.1371545	0.8414268
CYP17A1	Cytochrome P450 Family 17 Subfamily A Member 1	1.418715737	0.004554443	1.049381469	0.001851908	0.005875675	0.890819272	−0.03743256	0.9750525
INSR	Insulin Receptor	0.381745388	0.192710241	1.285645953	7.63097E-12	0.02287573	0.938544186	0.387068073	0.208446551
IGF1	Insulin Like Growth Factor 1	−0.673337332	0.124361875	−1.659370252	5.34224E-09	−0.646869143	0.0069179	−0.2747584	0.5370414
PLA2G4A	Phospholipase A2 Group IVA	−1.315828744	0.005668833	−1.883142744	5.15085E-14	−0.051607453	0.816803637	0.1022455	0.8456387

**Notes.**

OE ovarian endometriosis PE peritoneal endometriosis DIE deep infiltrating endometriosis Log2 FC Log2 fold change

We also noticed that both specific DEGs in OE and DIE were enriched in the pathways in cancer, though enriched genes were different. The enriched genes in the ‘pathways in cancer’ in DIE were COL4A3, COL4A6, PTGER3, RAD51, F2R, while OE seems to be associated with ‘pathways in cancer’ mainly by PI3K-Akt signaling pathway-related genes, such as FN1, GNG2, KIT, PTGS2, PDGFRA, LPAR1, CCND1, ITGA6, FGFR3, VEGFA, PIK3R1, FGFR2, MET, indicated by both microarray training datasets and RNA-sequencing validation dataset (Tables S3 and S4).

### PPI network construction and hub DEGs identification

**Figure 4:**
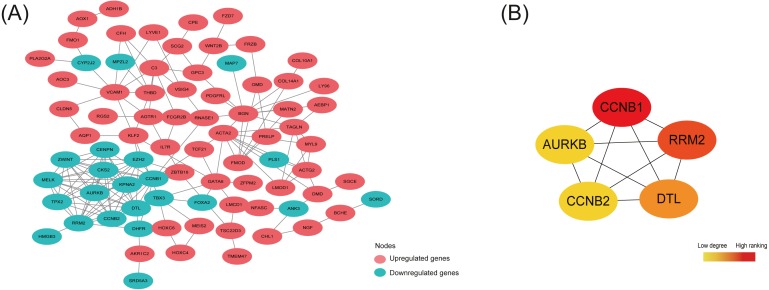
Protein-protein interaction (PPI) network construction and identification of hub nodes. (A) PPI network constructed by 148 shared DEGs among the merged OE dataset of GSE7305 and GSE7307, the individual PE dataset E-MTAB-694 and DIE dataset GSE25628. The red nodes represent upregulated genes and the green nodes represent downregulated genes. (B) Top 5 hub nodes (CCNB1, RRM2, DTL, CCNB2, AURKB) identified from the PPI network by using the Degree method in plugin Cytohubba. The nodes color changes gradually from yellow to red in ascending order according to the Degree ranking.

By analyzing 148 common DEGs in database STRING, a PPI network with 99 nodes and 178 edges was constructed and then visualized in Cytoscape (Fig. 4A). Furthermore, the top 5 hub nodes: CCNB1, CCNB2, RRM2, DTL, AURKB were identified from this PPI network by using the Degree method in plugin Cytohubba (Fig. 4B).

### Validation analysis of hub DEGs in GSE105764

As shown in Table 5, the expression trend of top 5 hub DEGs (CCNB1, CCNB2, RRM2, DTL, AURKB) was consistent in three subtypes—all under-expressed in ectopic lesions compared to eutopic endometrium, which was also further validated in the OE RNA-sequencing dataset GSE105764.

## Discussion

Endometriosis (EMs) was considered as a heterogeneous disease and its subtypes: ovarian endometriosis (OE), peritoneal endometriosis (PE), and deep infiltrating endometriosis (DIE) were likely to possess different aetiologies, which might require different diagnostic markers as well as treatments (Nisolle & Donnez, 1997; Gordts, Koninckx & Brosens, 2017). However, it is rare to see that all these three EMs subtypes were simultaneously included and analyzed in one study. To our best of knowledge, our article represents the first endeavor to analyze the common and specific molecular signatures among OE, PE, and DIE in a bioinformatic way based on effective microarray or RNA-sequencing datasets.

**Table 5:** The expression of the top 5 hub genes identified from the PPI network in microarray training datasets and the OE RNA-sequencing validation dataset GSE105764.

**Gene symbol**	**Gene description**	**OE RNA-sequencing dataset GSE105764**		**The merged OE dataset of GSE7305 and GSE7307**		**The individual PE dataset E-MTAB-694**		**The individual DIE dataset GSE25628**	
		**Log2FC**	***P*-Value**	**Log2FC**	***P*-Value**	**Log2FC**	***P*-Value**	**Log2FC**	***P*-Value**
RRM2	Ribonucleotide Reductase Regulatory Subunit M2	−2.545059091	5.65568E-06	−2.902398466	6.14787E-10	−3.521816066	1.01611E-08	−1.908309782	0.01636418
AURKB	Aurora Kinase B	−2.311429822	3.34652E-05	−1.42932862	3.39568E-09	−1.911232107	3.34734E-06	−1.0990377	0.039806461
CCNB1	Cyclin B1	−1.856061436	5.54985E-05	−2.207580173	3.967E-09	−2.161536326	3.96789E-06	−1.264483101	0.047858003
CCNB2	Cyclin B2	−1.825733268	0.000537883	−1.938433806	3.70498E-09	−2.689873382	7.46834E-07	−1.446885624	0.044121405
DTL	Denticleless E3 Ubiquitin Protein Ligase Homolog	−1.228039999	0.003406252	−1.757599775	1.88499E-08	−1.998898813	5.57114E-07	−1.372163345	0.032638654


**Notes.**

Log2 FC Log2 fold change OE ovarian endometriosis PE peritoneal endometriosis DIE deep infiltrating endometriosis

In this article, we conducted an integrative analysis of EMs mRNA microarray datasets belonging to three different subtypes: GSE7305 and GSE7307 of OE, E-MTAB-694 of PE, and GSE25628 of DIE. By analyzing DEGs between EU and EC samples, we found that 148 shared DEGs were common to three EMs subtypes, which were mainly involved in the pathways of arachidonic acid (AA) metabolism, vascular smooth muscle contraction, drug metabolism-cytochrome P450, complement and coagulation cascades, tyrosine metabolism revealed by KEGG enrichment analysis. Moreover, both the microarray training datasets and validation OE RNA-sequencing dataset showed that most of the genes in the top 1 enriched pathway—arachidonic acid (AA) metabolism pathway were activated in the EC tissues, except for CYP2J2.

In the arachidonic acid (AA) metabolism pathway, under the action of bioactive enzymes, substrate AA was catalyzed to generate endogenous eicosanoids such as prostaglandins (PGs), leukotrienes (LTs) and epoxyeicosatrienoic acids (EETs), which acted as mediators of various inflammatory disorders (Kuehl & Egan, 1980), including endometriosis (Benedetto, 1989; Monsivais et al., 2012; Sacco et al., 2012). PLA2G2A, a member of the phospholipase A2 family (PLA2) to catalyze the release of AA from membrane phospholipids, had been reported over-expressed in OE (Kocbek et al., 2015), DIE (Carrarelli et al., 2016) and PE (Lousse et al., 2010) ectopic lesions compared to normal or eutopic endometrium. The increased levels of PLA2G2A in endometriotic tissue would be responsible for providing AA for further PGs biosynthesis, such as PGE2, thus potentiating survival, migration, and invasion of endometriotic cells (Banu et al., 2008). The overexpression of PTGIS, functioning to convert prostaglandin H2 (PGH2) to prostaglandin I2 (PGI2), whose higher expression in the peritoneal fluid was observed in patients with endometriosis (Ylikorkala et al., 1984), had also been found a previous study (Monsivais et al., 2012). Under hypoxic conditions, PTGIS was reported to promote VEGF expression in human lung fibroblasts by producing PGI2 (Wang et al., 2013), whose enhanced production in ovarian endometrial cyst seemed to be associated with dysmenorrhea in endometriosis patients (Koike et al., 1992). GGT5 was responsible for converting leukotriene C4 (LTC4) to leukotriene D4 (LTD4), both of which were found with an increment of concentration in menstrual blood from patients with primary dysmenorrhea (Rees et al., 1987) and the highly selective LTD4 receptor antagonist had an inhibiting effect on endometriotic implant growth in rat endometriosis model (Kiykac Altinbas et al., 2015). However, our analysis also revealed that in the ectopic lesions, CYP2J2 was under-expressed, an enzyme of cytochrome P450 (CYP) superfamily to metabolize AA into EETs, which exerted an anti-inflammatory effect by various mechanisms (Shahabi et al., 2014). Taken together, the activated arachidonic acid (AA) metabolism and its corresponding products seemed to promote inflammation in ectopic lesions, thus facilitating the development of EMs.

Interestingly, the 5 hub nodes RRM2, AURKB, DTL, CCNB1, CCNB2 identified from the PPI network constructed by148 shared DEGs were all associated with cell cycle and mitosis, and down-regulated in the ectopic lesions in our analysis while always up-regulated in cancer tissues (Wang et al., 1997; Kolesar et al., 2009; Takashima et al., 2014; Kobayashi et al., 2015; Chieffi, 2018), thus promoting excessive proliferation, which suggested limited and controlled proliferative activity in EMs endometriotic lesions, distinct from cancerous proliferation feature. For example, RRM2, one of the subunits of ribonucleotide reductase complex providing precursors indispensable for DNA synthesis (Engström et al., 1985), was reported lowly expressed in EC compared to the EU in another genome-wide microarray study (Zafrakas et al., 2008), while its overexpression would enhance tumor angiogenesis and growth in multiple cancers (Zhang et al., 2009; Rahman et al., 2013). AURKB, a chromosomal passenger protein ensuring correct chromosome alignment and segregation in the mitosis (Kelly et al., 2010), was found low-expressed in OE ectopic tissues (Calcagno et al., 2011) whereas high-expressed in many tumors causing cell aneuploidy division (Giet, Petretti & Prigent, 2005). DTL, known as denticleless E3 ubiquitin-protein ligase homolog, was responsible for mediating the polyubiquitination and subsequent degradation of multiple regulators to ensure proper cell cycle progression (Higa et al., 2006), though seldom studied in EMs. However, the cyclin CCNB1, key regulator as well as CCNB2 in cell cycle controlling G2/M transition (Gong & Ferrell, 2010), were found up-regulated in ectopic tissue compared to eutopic endometrium in Tang et al. study (Tang et al., 2009), which was inconsistent with our analysis. This inconsistency might be explained by different detection methods and the intrinsic heterogeneity of EMs. Moreover, the phenomenon that most ectopic lesions contained sparse epithelial cells, which were abundant in eutopic endometrium and available to proliferate at a rapid speed, and mainly of stromal cells, whose multiplication rate was relatively mild, might result in the slow growth in ectopic tissue (Bulun et al., 2019).

Moreover, besides the common pathogenesis, we also explored the peculiar molecular signature in each subtype of EMs by the KEGG analysis of specific DEGs in different subtypes. For instance, most genes in the pathway of ovarian steroidogenesis were significantly up-regulated in EC in subtype OE while with no significant difference between EU and EC in the PE and DIE, which suggested ectopic tissue specificity and focal prominent aberrant hormonal environment in OE. Furthermore, the most up-regulated gene STAR in the pathway was proved to be correlated with the severity of OE and PE (Tian et al., 2009). That OE was regarded as an indicator of more severe pelvic and intestinal disease (Redwine, 1999) might be explained by this overactive ovarian steroidogenesis in OE since the steroid hormone was primarily produced in the ovarian and then reached endometriotic lesions via blood circulation and follicular fluid at the time of ovulation.

We also noticed that both specific DEGs in OE and DIE enriched in ‘pathways in cancer’, though enriched genes were different: DIE was associated with the risk of malignant transformation by enriched genes COL4A3, COL4A6, RAD51, F2R, PTGER3, while OE mainly by those PI3K pathway-related genes, such as FN1, GNG2, KIT, PTGS2, PDGFRA, LPAR1, CCND1, ITGA6, FGFR3, VEGFA, PIK3R1, FGFR2, MET. However, PE was more likely related to dysregulated peritoneal immune and inflammatory microenvironment, indicated by its specific DEGs enriched pathways, such as maladjusted cytokine-cytokine receptor interaction, leukocyte trans-endothelial migration, and natural killer cell-mediated cytotoxicity. Whether PE could be regarded as more ‘superficial’ or ‘slight’ disease primarily affected by peritoneal fluid factors, than OE and DIE mainly influenced by blood or ovarian factors (Koninckx, Kennedy & Barlow, 1998), still needs further investigation. Hence, the landscape of specific signatures in each subtype were not clear enough in our analysis due to a relatively small number of available microarray datasets as well as limited information provided by these datasets, especially the DIE dataset based on a platform with fewer probes, which would be modified by obtaining more RNA-sequencing datasets including all three subtypes and considerable samples in future analysis.

However, our analysis still has some limitations. Firstly, due to the lack of RNA-sequencing datasets for PE and DIE, validation analysis was only performed in subtype OE. Secondly, since the uterine endometrium undergoes molecular signature changes throughout the menstrual cycle (Talbi et al., 2006), different cycle phases might be one of the confounding factors: DIE samples were all collected during the proliferative phase, all PE samples and OE samples in GSE7305 were collected during either proliferative or secretory phase, while cycle phases of OE samples in GSE7307 were unknown. Comparing the gene expression in each subdivided cycle phase respectively would provide an improved perspective. Additionally, the contamination of normal tissue sometimes was unavoidable during the sampling process, which in a way would lead to potentially controversial results. For example, in our results, the significant overexpression of ovarian steroidogenesis related genes in OE while not in PE and DIE ectopic tissues may be explained by the intrinsic differences among subtypes and/or the mixture of normal ovarian tissues. This needs further laboratory investigation in the future.

## Conclusion

Through integrated bioinformatics analysis, we found that inflammation, especially arachidonic acid (AA) metabolism-related inflammatory process was the most common pathogenesis of OE, PE, and DIE. Besides, a slow and controlled proliferation in ectopic lesions was commonplace in these three EMs subtypes. Meanwhile, abnormal ovarian steroidogenesis was a prominent feature in OE; OE and DIE seemed to be at more risk of malignant development while PE tended to be more associated with dysregulated peritoneal immune and inflammatory microenvironment. All these findings could deepen our understanding of the common and specific molecular events in different subtypes in endometriosis.

##  Supplemental Information

10.7717/peerj.8730/supp-1 Table S1 Clinical characteristics of patients with peritoneal endometriosis (PE) in E-MTAB-694
Left to right: Patient I.D. (n=19), age in years (mean = 34.32 ± 7.6 years); stage of endometriosis peritoneal lesion according to the American Fertility Society [AFS] classification; menstrual cycle phase according to Materials and Methods in original article DOI: 10.1177/1933719112451147; tissues analyzed; (+, available; −, not available).

10.7717/peerj.8730/supp-2 Table S2 Clinical characteristics of patients with Deep Infiltrating Endometriosis (DIE) in GSE25628
Left to right: Patient I.D. (n=8), age in years (mean = 31.5 ± 5.2 years); stage of endometriosis deep infiltrating lesion according to the American Fertility Society [AFS] classification; menstrual cycle phase according to Materials and Methods in original article DOI: 10.1002/jcp.24358; tissues analyzed; (+, available).

10.7717/peerj.8730/supp-3 Table S3 All the significantly enriched KEGG pathways of 729 specific DEGs in OE Abbreviations: Log2 FC, Log2 fold change; OE, ovarian endometriosis.

10.7717/peerj.8730/supp-4 Table S4 The expression of genes enriched both in ‘pathways in cancer’ and ‘PI3K-Akt signaling pathway’ in OE in the microarray training dataset and RNA-sequencing validation dataset GSE105764
Abbreviations: Log2 FC, Log2 fold change; OE, ovarian endometriosis.

10.7717/peerj.8730/supp-5 Figure S1 Data preprocessing and normalization (A), (B), (C), Boxplot of gene expression values after normalization in the merged OE dataset of GSE7305 and GSE7307, the individual PE dataset E-MTAB-694 and DIE dataset GSE25628. Boxplots represent the mean ± interquartile range, with whiskers extending to the minimum and maximum value. The Abscissa axis represents samples; the vertical axis represents gene expression value; the black horizontal line represents the median of the gene expression value for each sample; the green boxes represent EU samples; the red boxes represent EC samples. OE, ovarian endometriosis; PE, peritoneal endometriosis; DIE, deep infiltrating endometriosis; EC, ectopic lesions; EU, eutopic endometrium.

## Additional Information and Declarations

### Data Availability

The following information was supplied regarding data availability: Raw data are available at NCBI GEO: GSE7305, GSE7307, GSE25628, and GSE105764, and at ArrayExpress: E-MTAB-694.
