# Supplementary figures and images for: Common and specific gene signatures among three different endometriosis subtypes
Source: PeerJ. 2020 Mar 5;8:e8730. doi: 10.7717/peerj.8730 (PMC7060988; doi:10.7717/peerj.8730)

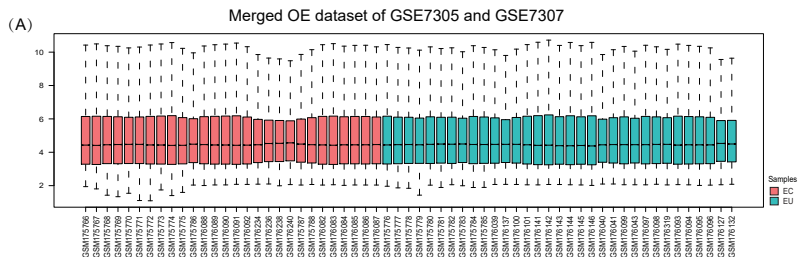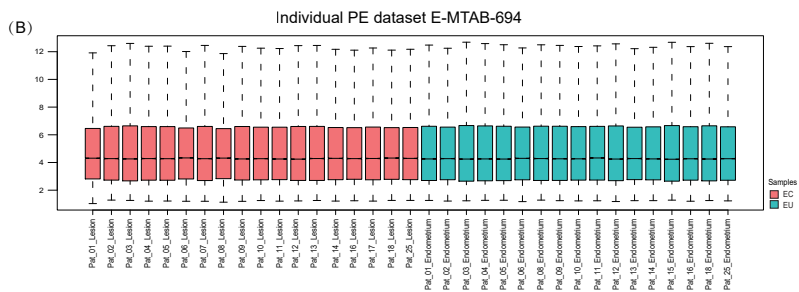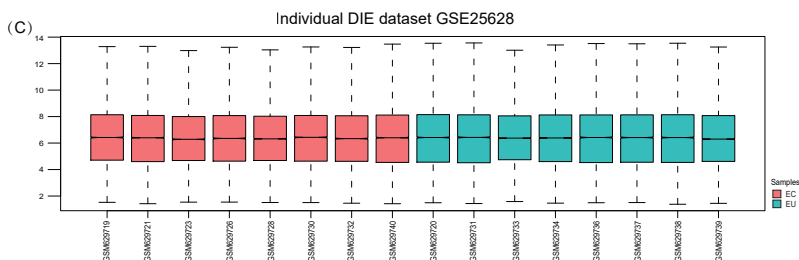

Supplement: Figure S1 — (A), (B), (C), Boxplot of gene expression values after normalization in the merged OE dataset of GSE7305 and GSE7307, the individual PE dataset E-MTAB-694 and DIE dataset GSE25628. Boxplots represent the mean ± interquartile range, with whiskers extending to the minimum and maximum value. The Abscissa axis represents samples; the vertical axis represents gene expression value; the black horizontal line represents the median of the gene expression value for each sample; the green boxes represent EU samples; the red boxes represent EC samples. OE, ovarian endometriosis; PE, peritoneal endometriosis; DIE, deep infiltrating endometriosis; EC, ectopic lesions; EU, eutopic endometrium. [file peerj-08-8730-s005.pdf]
